# Close Encounters of the Third Domain: The Emerging Genomic View of Archaeal Diversity and Evolution

**DOI:** 10.1155/2013/202358

**Published:** 2013-11-19

**Authors:** Anja Spang, Joran Martijn, Jimmy H. Saw, Anders E. Lind, Lionel Guy, Thijs J. G. Ettema

**Affiliations:** Department of Cell and Molecular Biology, Science for Life Laboratory, Uppsala University, P.O. Box 596, 75123 Uppsala, Sweden

## Abstract

The Archaea represent the so-called Third Domain of life, which has evolved in parallel with the Bacteria and which is implicated to have played a pivotal role in the emergence of the eukaryotic domain of life. Recent progress in genomic sequencing technologies and cultivation-independent methods has started to unearth a plethora of data of novel, uncultivated archaeal lineages. Here, we review how the availability of such genomic data has revealed several important insights into the diversity, ecological relevance, metabolic capacity, and the origin and evolution of the archaeal domain of life.

## 1. Introduction

The description of the three (cellular) domains of life—Eukarya, Bacteria, and Archaea—by Carl Woese and George Fox [1] represents a milestone in the modern era of microbiology. In particular, using phylogenetic reconstructions of the small-subunit (16S or 18S) ribosomal RNA gene, Woese discovered that microscopically indistinguishable prokaryotes are not a homogeneous assemblage but are comprised of two fundamentally different groups of organisms: Eubacteria (later Bacteria) on one side and an additional life form referred to as Archaebacteria (later Archaea) on the other side [1]. Though not immediately accepted by the scientific community, this finding was early on supported by Wolfram Zillig through his studies on DNA-dependent RNA polymerases, as well as by Otto Kandler investigating “bacterial” cell walls [2]. Indeed, a subset of prokaryotic organisms subsequently assigned to Archaea was found to harbor DNA-dependent RNA polymerases that bore more similarity to those of eukaryotes, and to contain proteinaceous cell walls that lack peptidoglycan as well as cell membranes composed of L-glycerol ether lipids with isoprenoid chains instead of D-glycerol ester lipids with fatty acid chains [3–6]. Since then, further investigation of cellular characteristics of archaea has revealed that this domain of life contains eukaryotic-like information-processing machineries [7–14]. These findings were later supported by genome sequences and comparative analyses of genes coding for replication, transcription, and translation machineries as well as by protein crystal structures [15–21]. Additionally, some archaeal lineages were shown to contain homologs of eukaryotic cell division and cytoskeleton genes as well as histones and seem to express a chromatin architecture similar to eukaryotes [22–28]. In contrast to information-processing and cell division genes, archaeal operational systems (energy metabolism, biosynthesis pathways, and regulation) often appear to be more closely related to bacteria [29].

Based on phylogenetic reconstructions of the evolutionary history of 16S rRNA genes, the domain Archaea was originally divided into two major phyla: the Euryarchaeota and Crenarchaeota [30], which were separated by a deep split and thought to comprise only extremophilic (thermophilic, halophilic, and acidophilic) as well as methanogenic organisms. However, novel culture-independent and high-throughput sequencing techniques have recently uncovered a huge diversity of so far uncharacterized microorganisms on Earth as well as the ubiquitous occurrence of archaeal species [31–33]. Many of these novel archaeal groups are responsible for important ecological processes and are only distantly related to established lineages within Cren- and Euryarchaeota [31, 32, 34–39]. For example, the acquisition of genome sequences from novel archaeal representatives has led to the proposal of several additional archaeal phyla (including Nanoarchaeota, Korarchaeota, Thaumarchaeota, Aigarchaeota, and Geoarchaeota) [40–46] and the investigation of uncultivated archaea using single cell genomics has already started to add new insights into the phylogenetic diversity of the Third Domain of life and necessitates the definition of additional lineages of high taxonomic rank including novel potential phyla and superphyla [33, 39] (see also below). Furthermore, the investigation of the metabolic potential of these novel organisms has provided fundamentally new insights into major biogeochemical nutrient cycles. Indeed, archaea are now recognized as key players in various biogeochemical processes [47]. For example, the perception of the global nitrogen cycle has been deeply altered by discovering that the ability to gain energy solely from ammonia was not limited to a few bacteria but also included the ammonia-oxidizing Thaumarchaeota [48, 49]. Archaea also appear to play a significant role in the carbon cycle, since, in addition to all known methanogenic organisms on Earth, they also encompass anaerobic methane oxidizing archaea (ANME lineages 1–3) [50]. 

The study of archaeal genomes and diversity is also of considerable importance for a better understanding of eukaryotic evolution. Indeed, the discovery of eukaryotic features in archaea [10] has initiated a new basis for addressing the origin of eukaryotes [51–54]. Interestingly, recent phylogenetic analyses of universal proteins have suggested that eukaryotes might have evolved from a *bona fide* archaeal lineage that forms a sister-lineage of or a lineage emerging from within the TACK-superphylum comprised of Thaum-, Aig-, Cren-, and Korarchaeota [55–58].

 Below we give a contemporary overview of how recent developments in archaeal genomic research have contributed to revealing new insights into the diversity, ecological relevance, metabolic capacity, and the origin and evolution of the archaeal domain of life. 

## 2. The Methanogenic Nature of Archaea

**Figure 1:**
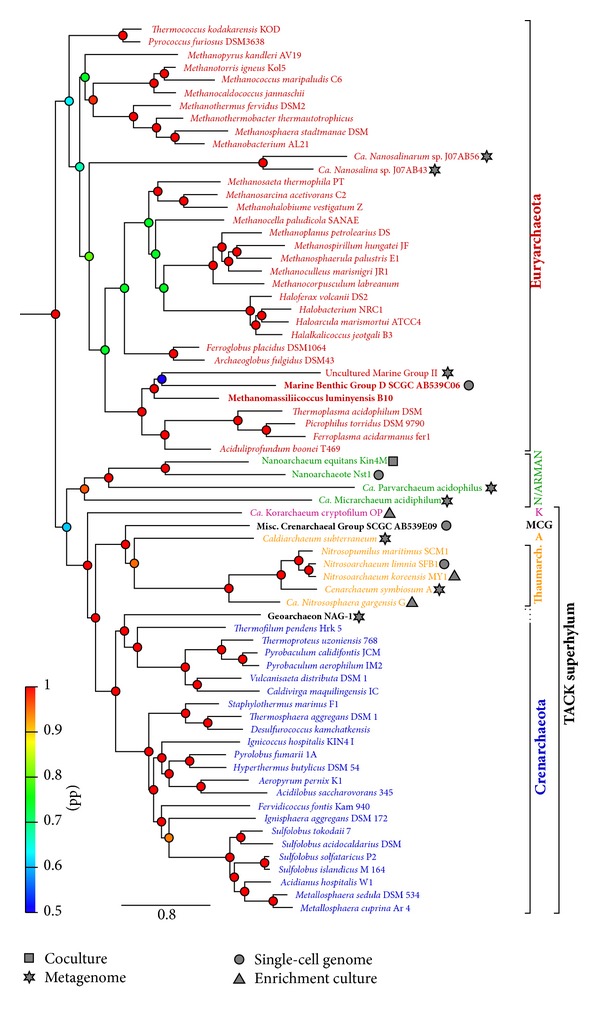
Bayesian phylogeny of 80 representative archaeal species. BLAST databases containing the proteome of 6 new archaeal genomes were retrieved from NCBI (in bold font on the tree): *Methanomassiliicoccus luminyensis* B10 (acc. no. CAJE01), MCG SCGC AB539E09 (acc. no. ALXK01), Marine Benthic Group D (MBGD) SCGC AB539N05, AB539C06, and AB540F20 (acc. no. ALXL01, AOSH01, and AOSI01, resp.). Protein sequence alignment from the 57 clusters in the discFilter 15 p dataset from [70] for which eukaryotes were removed were used as an input to psi-blast, with the six new proteomes as a database. Orthologs were retrieved as in [70]. For the three MBGD strains, one composite set of orthologs was constituted by using the most complete one (AB539C06) whenever possible and complementing with sequences from the other two if available. Orthologous genes selection, alignment, trimming, and concatenation were performed as in [70] resulting in a 15,069 amino-acid alignment. Four chains of Bayesian phylogenies were run with Phylobayes [71], under the CAT-Poisson model, running for approximately 10000 generations and discarding half as a burn-in. The tree was rooted with bacteria. Posterior probabilities (pp) are represented by colored dots on the nodes, with support values coloured according to the depicted heat-map colour scheme. The scale represents the number of substitutions per site. Species are colored according to the following: red, Euryarchaeota; green, Nanoarchaeota (N) and ARMAN; pink, Korarchaeota (K); black, Misc. Crenarchaeal Group (MCG); orange, Thaumarchaeota and Aigarchaeota (A); blue, Crenarchaeota. The DNA collection method, if different from pure culture, is indicated by a symbol next to the organism name: square, coculture; star, metagenome; circle, single-cell genome; triangle, enrichment culture.

The scientific community that addressed questions about prokaryotic energy metabolism on the early Earth or in hydrothermal vent systems [59] has proposed that methanogenesis and/or acetogenesis most likely represent ancient metabolic pathways [60–62]. Evidence for the biological production of methane as early as 3.46 Gyr ago supports these scenarios [63]. However, phylogenetic evidence placing methanogens at the base of the archaeal tree is limited and disputed. Depending on the outgroup and phylogenetic methods used, many recent analyses find either members of the Nanohaloarchaea, Nanoarchaeota, ARMAN-lineages, and/or *Thermococcales* as earliest (eury-)archaeal branches [55, 64, 65]. The latter observation is consistent with results from a base and amino acid composition analysis, which indicated that last archaeal common ancestor (LACA) was a hyperthermophilic organism [66]. The placement of *Methanopyrus kandleri* as the most basal branch of archaea in some of the earliest phylogenetic analyses can most likely be attributed to long-branch attraction (LBA) artifacts [67]. Notably, in recent phylogenetic analyses that include novel archaeal single cell genomes, Euryarchaeota form a sister group to other archaeal phyla rather than representing an early diverging lineage (Figure 1) [33]. Furthermore, gene content comparisons of extant archaeal lineages and reconstruction of the putative genetic repertoire of the LACA do not support methanogenesis as the earliest archaeal metabolism [57, 68]. In contrast, only one study has so far placed the root of archaea within a methanogenic order [69] and thus favors a methanogenic origin of the Third Domain of life. Gene content comparisons and network analyses that include novel archaeal single-cell amplified genomes (SAGs) Could potentially help to further investigate the metabolic gene repertoire of the archaeal ancestor.

Whereas the origin of methanogenic pathways that include a multitude of specific genes and cofactors is not fully resolved yet [72], it appears that several later emerging euryarchaeal lineages have lost their methanogenic lifestyles. Thus, as already noted more than a decade ago, methanogens comprise a paraphyletic group separated by nonmethanogenic euryarchaeal lineages such as the *Thermoplasmatales*, Haloarchaeota, and *Archaeoglobales* [73]. Interestingly, a novel methanogenic archaeal lineage has been described recently that is distantly affiliated with cultivated *Thermoplasmatales* including *Aciduliprofundum* sp. [74, 75]. This suggests that the last common ancestor of *Thermoplasmatales* was a methanogen and the capability to reduce methane has been independently lost several times along some branches within this group [76] or, albeit less likely, that some lineages within the *Thermoplasmatales* have regained genes for methane production.

A single acquisition of a plethora of genes (>1000) from a bacterial donor has recently been put forward as explanation for the transition from a methanogenic ancestor to aerobic heterotrophic Haloarchaeota [77]. A possible driving force for this massive gene transfer might have been a syntrophic relationship between a methanogenic recipient and a bacterial donor. However, the exact donor lineage could not be determined: the acquired genes bear conflicting phylogenetic signals, supposedly due to prevalent gene transfers between different bacterial species. So far, the origin of alternative energy metabolisms in other non-methanogenic euryarchaeal lineages that evolved from methanogenic ancestors has not been addressed properly. However, comparative genomics suggests that several of these lineages have retained specific genes that trace back to the methanogenic nature of their ancestor (e.g., *Archaeoglobus*) [78] and might point to a rather transient transition.

## 3. Phylogeny of New Archaeal Phyla and Lineages

In recent years, several new archaeal lineages have been identified and subjected to whole genome or metagenomic sequencing. Based on phylogenetic analyses of available genomic data, some of these lineages have been proposed to represent novel archaeal phyla. Yet, some of these claims have been challenged or falsified in follow-up studies. Below, we give an overview of several such examples.

The candidate phylum Nanoarchaeota has initially been proposed on basis of the extremely divergent 16S rRNA sequence of the small parasitic cells of *Nanoarchaeum equitans* growing attached to the cell surface of *Ignicococcus hospitalis* [41]. Several subsequent and more comprehensive phylogenetic analyses as well as the finding of potentially ancestral genomic features (e.g., split tRNA genes) have provided support for the initial assignment of this tiny archaeal cells to a separate ancient archaeal phylum [65, 79, 80]. Yet, in contrast, other phylogenetic and comparative analyses testing the taxonomic position of *N. equitans* have suggested that Nanoarchaeota might rather represent a fast-evolving euryarchaeal lineage related to *Thermococcales* [81]. Genomic data from additional “nanosized” archaea (*Ca. *Parvarchaeum acidophilus ARMAN-4 and *Ca.* Micrarchaeum acidiphilum ARMAN-2) [82] as well as of a novel deep-branching member of Nanoarchaeota (Nst1) [83] have enabled a revision of phylogenetic reconstructions and genome comparisons. Although some of these analyses suggest that Nanoarchaeota and *Ca. *Parvarchaeum acidophilus are monophyletic, the placement of these groups in the archaeal tree remains unclear and is strongly dependent on dataset and phylogenetic methods used [64, 83]. For example, in our phylogenetic reconstructions Nanoarchaeota (including ARMAN-lineages) represent a sister clade of the TACK superphylum (Figure 1), although the support for this clade is low. In a recent study by Rinke et al. [33], the Nanoarchaeota (including all ARMAN strains) were grouped together in the newly proposed superphylum DPANN with two novel groups, DSEG and pMC2A384 (designated “Aenigmarchaeota” and “Diapherotrites”, resp.), as well as the Nanohaloarchaea (see also below). Given that the phylogenetic methods employed by Rinke and coworkers do not accommodate rate heterogeneity across taxa, the proposed grouping of Nanoarchaeota with these archaeal clades has to be taken with care and the exact position of Nanoarchaeota still remains an unresolved question.

The Nanohaloarchaea represent yet another archaeal lineage comprised of small cells and with unresolved phylogenetic position. Based on both 16S rRNA gene and concatenated ribosomal protein phylogenies, this group was suggested to comprise a deep lineage of Haloarchaeota [84]. However, only euryarchaeal sequences were included in these maximum-likelihood (ML) analyses. Depending on the phylogenetic method and evolutionary model used, we obtained contradictory results for the phylogenetic position of this group. Whereas ML analyses tend to recover Nanohaloarchaea as earliest branching archaeal lineage (e.g., see above), a phylogenetic reconstruction using Bayesian methods (and the CAT model [71]) place this lineage within Euryarchaeota, but the exact position could not be resolved with high confidence (Figure 1). Results obtained with Bayesian methods using the CAT model might provide a better approximation of the position of Nanohaloarchaea, as this model accounts for rate variations across sites. As such, the early divergence of Nanohaloarchaea that is observed in ML-based methods is likely caused by LBA artifacts. However, novel phylogenetic analyses including the improved archaeal taxon sampling of Rinke et al. suggest that Nanohaloarchaea form a distinct lineage within the proposed superphylum DPANN and are not closely related to Euryarchaeota [33].

It will be interesting to further address the position of these organisms in the archaeal tree to be able to elucidate whether the adaptation to halophily has evolved only once in archaea or is due to convergence in Halo- and Nanohaloarchaea. The latter has received initial support from comparative genome analyses, which have revealed that each of these two archaeal groups seems to harbor diverse unique features including distinctive amino acid compositions to accommodate high salt conditions [84]. It might also be of value to address the effect of these novel genome sequences on the results obtained in the analysis of Nelson-Sathi et al. studying the origin of Haloarchaeota from a methanogenic ancestor [77]. 

Another novel archaeal phylum comprises the abundant and ecologically important ammonia-oxidizing archaea (AOA). On the basis of comparative genomics and phylogenetic analyses based on concatenated ribosomal proteins that were rooted with eukaryotes, Brochier-Armanet and coworkers proposed that “mesophilic crenarchaeota” constitute the novel deep branching archaeal phylum Thaumarchaeota [42, 85]. Additional comprehensive phylogenetic analyses including additional members of this group, as well as the discovery of a distinctive set of informational processing genes involved in replication, transcription, and translation as well as DNA repair and cell division machineries, have provided further support for the independent status of the Thaumarchaeota [44]. For example, in contrast to Crenarchaeota, Thaumarchaeota share several characteristics with Euryarchaeota and Korarchaeota including the presence of DNA polymerase D, histones, and cell division protein FtsZ. Furthermore, they contain putative “ancestral” features absent from Cren- or Euryarchaeota but common in Bacteria and eukaryotes (e.g., presence of an unsplit gene encoding DNA polymerase subunit A, and toposimerase IB as well as the absence of ribosomal protein LXa) [20, 44, 85]. The distinct nature of Thaumarchaeota has been accepted by many authors [45, 46, 86, 87] although the taxonomic borders of this phylum are still difficult to delineate and might only be resolved when genomes of uncultivated early branching lineages are made available. The early emergence of Thaumarchaeota in these phylogenetic reconstructions using eukaryotes as outgroup was initially assumed to indicate the ancient nature of this phylum [42, 44]. However, several recent phylogenetic analyses have recovered a monophyletic group of Thaum-, Aig-, Cren, Korarchaeota, and eukaryotes (with varying relationships in between these groups) to the exclusion of Euryarchaeota, which indicates that eukaryotes emerge from within the Archaea [55, 56, 88]. Thus, eukaryotes cannot be used as valid outgroup for the rooting of archaeal phylogenies [54].

Another lineage that emerges as a separate branch in the archaeal tree is comprised of the so-called Hot Water Crenarchaeotic Group I (HWCG I), members of which have been detected in diverse hydrothermal environments but have not yet been cultivated [89, 90]. Until recently, the sole representative with a sequenced genome in this group was *Ca.* Caldiarchaeum subterraneum, whose composite genome has been obtained from a metagenomic library of a microbial mat in a subsurface geothermal water stream [45]. The investigation of its genome sequence has revealed the presence of components of the eukaryotic ubiquitin-like protein modifier system previously not detected in archaea or bacteria. This unique trait, as well as comparative genomics and phylogenetic analyses of concatenated protein sequences, suggested that this organism and other members of HWCG I might constitute a novel phylum (Aigarchaeota), distinct from both Thaum- and Crenarchaeota [45]. However, due to the presence of a set of informational processing genes most similar to Thaumarchaeota [45] and the highly supported monophyletic grouping of these two lineages in diverse phylogenetic analyses (e.g., see Figure 1), the separation of Thaum- and Aigarchaeota into two distinct phyla is still debated [45, 55, 56, 64, 91, 92].

Uncultivated archaea belonging to the so-called Miscellaneous Crenarchaeotal Group (MCG) (e.g., [39]) have been suggested to represent additional members of Aigarchaeota [55]. Recently, the first single-cell genome of a member of this group has been obtained and phylogenetic analyses of concatenated conserved single copy genes placed the MCG-archaeon as a lineage in between Thaum- and Aigarchaeota [97]. However, our analyses rather suggest that MCG emerges prior to the Thaum/Aigarchaeota (Figure 1). The availability of additional genome sequences of members of this group as well as the comparison of informational processing marker genes [44] of MCG-archaea with other available archaeal genomes might help both to resolve their phylogenetic placement and to determine whether MCG-archaea comprise a separate archaeal phylum [39]. 

Geoarchaeota represents yet another recently proposed archaeal phylum, which is proposed to emerge as a basal lineage of Crenarchaeota and includes the so-called novel archaeal group I (NAG-1) detected in acidic ferric iron mats from Yellowstone National Park [46, 98]. NAG-1 organisms thrive in hot (60–78°C) acidic mats rich in iron and are suggested to grow heterotrophically from simple carbon compounds. Though not yet enriched in culture, nearly full-length genome sequences of members of this group have been obtained from a *de novo *metagenome assembly. The description of this lineage as a separate phylum was based on phylogenetic analyses of concatenated ribosomal proteins and 16S/23S rRNA genes as well as on its specific set of informational processing genes with features in common with either Crenarchaeota or Thaum- and Aigarchaeota [46]. However, our analyses, based on a larger dataset, place Geoarchaeota as an early branching lineage of the crenarchaeal order Thermoproteales (Figure 1). This observation is confirmed by Rinke et al., who sequenced six additional NAG-1-related strains [33]. Indeed, detailed phylogenetic analyses, as well as comparative assessment of the NAG-1 composite genome, seem to refute the phylum-level status of NAG-1 (Guy, L., Spang, A., Saw, J.H. and Ettema, T.J.G., unpublished observation).

## 4. Archaea and the Origin of Eukaryotes

The origin of the eukaryotes remains one of the major unanswered questions in modern biology, and archaea have recently reclaimed the spotlights in heated discussions entailing this enigmatic event. A central issue in this discussion entails the placement of the root within the Tree of Life, as it has a fundamental effect on any hypothesis on the origin of eukaryotes. Whereas diverse competing hypotheses have been put forward in the past, no consensus has been reached on this topic so far. For instance, several studies, including a recent network analysis, place the root between Archaea and Bacteria [99–104]. This view is in agreement with both the observed fundamental differences distinguishing the bacterial and archaeal domains as well as with the geological record. In contrast, studies that were based on transmission analyses or the distribution of indels in protein sequences suggested a rooting within the bacterial domain [105–107], whereas a root in the archaeal domain has been proposed based on analyses of protein folds or the evolution of the tRNA molecules [108, 109]. Yet other hypotheses state that LUCA was a eukaryotic-like organism [110, 111]. Certainly, in order to reach a consensus on this controversial discussion, additional data and analyses are needed. Bearing the uncertainty of the placement of the root in the Tree of Life in mind, we will present current hypotheses on the origin of eukaryotes below, by providing a short review on the most commonly proposed scenarios.

**Figure 2:**
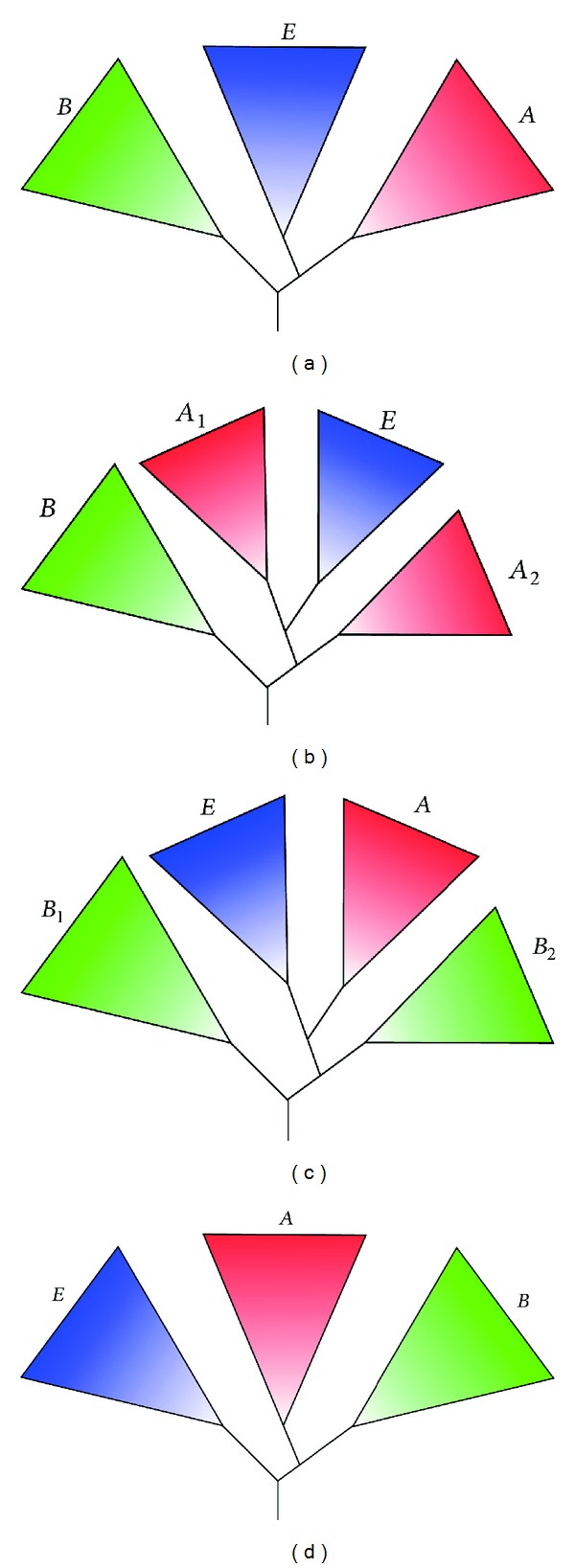
Overview of theories regarding the origin of the eukaryotic nuclear lineage. (a) The classical, Woesean three domains of life tree in which the nuclear lineage vertically evolved from the archaea-eukaryote common ancestor. (b) The fusion tree in which the nuclear lineage originated from the archaeal partner in the fusion. Depending on which fusion model, the archaeal parent's lineage (*A*_1_) was either part of the euryarchaeota [SET [93], original syntrophy hypothesis [94], hydrogen hypothesis [95] or alternative syntrophy hypothesis [51]], the Crenarchaeota (eocyte hypothesis) [96], or the TACK superphylum (PhAT) [58]. “*A*_2_” represents all archaea not directly affiliated with “*A*_1_.” (c) The neomuran tree in which the eukaryotic and archaeal lineage (combined referred to as “neomurans”), evolved vertically from ancestor shared with actinobacteria (*B*_2_) as a result of the loss of bacterial-type cell wall (the neomuran revolution). *B*_1_ represents all bacteria not directly affiliated with *B*_2_. (d) The eukaryote-early tree, which suggests that the last common universal ancestor was more eukaryote-like than prokaryote-like.

Even though a wide variety of incompatible theories have been suggested regarding the origin of the eukaryotic cell, three aspects are now largely accepted: (i) the last eukaryotic common ancestor (LECA) contained mitochondria, (ii) eukaryotic genomes are chimeric; whereas informational genes are of archaeal descent, many metabolic genes are derived from Bacteria, and (iii) eukaryotes complement a set of proteins not found in either Archaea or Bacteria, the eukaryotic signature proteins (ESPs). Beyond this, the picture becomes blurry. Currently, two major questions are of interest. What was the nature of the cell that was host in the mitochondrial endosymbiosis and when did cellular complexity evolve, before (complexity-first) or after (mitochondria-first) mitochondrial endosymbiosis? From this perspective, theories on eukaryogenesis can be divided into two categories. In the first scenario, the host was a protoeukaryote and complexity evolved first. This theory, often referred to as the “archezoa hypothesis” [112, 113], fits with the three domains tree of life model in which eukaryotes vertically evolved from the archaea-eukaryote common ancestor (Figure 2(a)). In the second scenario, the host was a prokaryote and the acquisition of the mitochondria likely triggered the evolution of cellular complexity. The latter are often referred to as “fusion” hypotheses [51, 58, 93, 95, 96] and these are generally incompatible with the classical three domains model. Rather, in these models, Bacteria and Archaea are the primary domains of life and eukaryotes a secondary, or derived, domain of life (Figure 2(b)). Theories that fit neither of these categories exist as well. These include the neomuran hypothesis [114] (Figure 2(c)), the PVC hypothesis [115–118], virus-assisted eukaryogenesis [119–122], and a hypothesis suggesting a eukaryote-like universal common ancestor [110] (Figure 2(d)). In order to choose the correct category with high confidence, evidence is needed in the form of protoeukaryote intermediate lineage's descendants (“missing links”). Unfortunately, for either category, none has been found so far. Whereas the archezoa theory has lost much support ever since remnants of mitochondria were found in the previously thought archezoa (for review, see [123]), the fusion theory has slowly been gaining favor. Initially lightly supported by ribosomal structural features [124] and an 11-amino acid insertion in EF-1*α*/EF-Tu [125, 126] shared between eocytes (Crenarchaeota) and eukaryotes to the exclusion of other prokaryotes, it has now received strong support from phylogenomic [55, 56, 70, 88, 127, 128] and gene similarity network analyses [129]. In addition, a large number of ESPs has been found in Archaea, in particular within the recently proposed TACK superphylum [55]. Examples include actin [53, 130], tubulin [28], H3/H4-type histones [55], ESCRT-III [24, 25, 131], and components of the ubiquitin modifier system [45]. Fusion models can be subdivided based upon the nature of the end-product of the “fusion”. In amitochondriate models the symbiosis results in a eukaryotic progenitor lacking mitochondria. They are similar to the archezoa theory in the sense that the origin of eukaryotes and the origin of mitochondria are separate events. These include the serial endosymbiosis theory (SET) [93], the original syntrophy hypothesis [94], and the eocyte hypothesis [96]. In mitochondriate models, the end product is a eukaryotic progenitor containing mitochondria. Here, the origin of eukaryotes and mitochondria are one and the same. These include the hydrogen hypothesis [95], the alternative syntrophy hypothesis [51] and the recently proposed phagocytosing archaeon theory [53, 58]. With exception of the eocyte hypothesis, all fusion theories suggest an archaeal host. Based on extensive, in-depth phylogenomic studies, the archaeal host most likely emerged from within the TACK superphylum [55, 56, 70]. Interestingly, out of all TACK phyla, a sister relationship between the Korarchaeota and eukaryotes was retrieved with significant phylogenetic support [56, 70]. Even though this placement could be a taxon sampling artifact (Korarchaeota are represented by a single, deep rooting taxon), it could also indicate that eukaryotes are affiliated with an unidentified lineage distantly related to Korarchaeota. Genomically unexplored lineages such as DSAG (Deep Sea Archaea Group), MHVG (Marine Hydrothermal Vent Group), and AAG (Ancient Archaea Group) are likely candidates [55, 70].

## 5. Genomic Assessment and Taxonomic Classification of Archaeal Diversity

Recent progress in genomic sequencing technologies and cultivation-independent methods has started to unearth a plethora of novel, uncultivated archaeal lineages. The availability of such genomic data has revealed several important insights into the diversity, ecological relevance, metabolic capacity, and the origin and evolution of the archaeal domain of life. Several new archaeal lineages have been obtained by means of metagenomics approaches, such as sequencing of enrichment cultures or environmental samples. Examples of the former include the first korarchaeal genome [43] and several of the available thaumarchaeal genomes (e.g. [132, 133]). Archaeal genomes that have been retrieved from metagenomic datasets include the first thaumarchaeal genome (*Ca.* Cenarchaeum symbiosum [134]), the genome of the proposed Aigarchaeon *Ca.* C. subterraneum [45], the proposed Geoarchaeon NAG-1 [46], representatives of the Nanohaloarchaea [84], several ARMAN lineages that were part of an acid mine drainage microbial community [82], and a genome derived from a representative of the uncultivated marine group II euryarchaeota [135] (Figure 1). More recently, a number of studies have employed single cell genomic approaches to probe the genetic diversity of uncultivated archaea. For example, Lloyd and coworkers have reported the first genomic data of a representative of the Miscellaneous Crenarchaeal Group (MCG) and of members of the Marine Benthic Group D that were isolated from marine sediments and speculate that these lineages are involved in the degradation of detrital proteins [97] (Figure 1). Another large scale study that aimed at uncovering the coding potential of so-called “microbial dark matter” using single cell genomics approaches reported several genome sequences of cells that potentially represented novel phylum-level archaeal lineages, including the members of the uncultured DSEG and pMC2A384 clades, designated Aenigmarchaeota and Diapherotrites, respectively [33]. A combination of single cell genomics and metagenomics has been used to sequence the genome of the thaumarchaeon *Ca.* Nitrosoarchaeum limnia SFB1 [136].

Obviously, single cell and metagenomics-oriented projects will continue to probe the existing archaeal diversity during the coming years, and most likely, the availability of genomic data will reveal interesting insights into novel characteristics and the diversity within the Third Domain of life. In addition, the availability of such genomic data is likely to trigger discussions regarding the higher-order taxonomic classification of the major archaeal lineages. To many (micro-)biologists, it would appear that the archaeal domain is far less diverse than the bacterial domain. A reason for this could be, for instance, the discrepancy in assigned or proposed phyla, which ranges from a handful in Archaea, to well over a hundred in Bacteria. But is it really fair to say that the bacterial domain of life is more diverse than that of the Archaea? Whereas bacterial phyla generally have been assigned based on the diversity of the 16S rDNA gene sequence, archaeal taxonomy is largely founded on historic grounds, that is, adhering to the classical Cren-Euryarchaeota dichotomy (sensu Woese [30]). Only during the past decade, a handful of additional archaeal phyla have been proposed based on genome sequencing, such as the Nano-, Kor-, and Thaumarchaeota and a few other lineages that may or may not represent phylum-level archaeal clades (also see above). Yet, the majority of archaeal species that have been sequenced in recent years have been assigned to the phyla Cren- or Euryarchaeota, each of which now comprise genetically distinct groups, which differ in terms of metabolic capacity, lifestyle, and environmental distribution. In light of this and of the abovementioned “superficial” imbalance in bacterial versus archaeal diversity, one could argue that a revision of archaeal higher-order taxonomy is in place. The suggestion to bring order into archaeal systematics was recently put forward [92], but thus far, a framework as to how novel phyla and/or superphyla should be defined is debated. Nevertheless, to be able to fully appreciate the overall archaeal diversity and compare it to the diversity observed within the bacterial domain of life, a reappraisal of the archaeal taxonomy, whether it will be at the level of rRNA genes, large datasets of concatenated protein sequences, genome content, or gene networks analyses, seems to be a *conditio sine qua non*.

## Abbreviations

ANME: Anaerobic methanotrophic archaea
LACA: Last archaeal common ancestor
LBA: Long-branch attraction
MCG: Miscellaneous Crenarchaeotal Group
ML: Maximum-likelihood
SAGs: Single-cell amplified genomes.
